# Comparative Analysis of Virulence and Molecular Diversity of *Puccinia striiformis* f. sp. *tritici* Isolates Collected in 2016 and 2023 in the Western Region of China

**DOI:** 10.3390/genes15050542

**Published:** 2024-04-25

**Authors:** Tesfay Gebrekirstos Gebremariam, Fengtao Wang, Ruiming Lin, Hongjie Li

**Affiliations:** 1 The National Engineering Laboratory of Crop Molecular Breeding, Institute of Crop Sciences, Chinese Academy of Agricultural Sciences, Beijing 100081, China; tesfaygebre2009@gmail.com; 2 Tigray Agricultural Research Institute, Mekelle P.O. Box 492, Ethiopia; 3 State Key Laboratory for Biology of Diseases and Insect Pests, Institute of Plant Protection, Chinese Academy of Agricultural Sciences, Beijing 100193, China; wangfengtao@caas.cn; 4 Institute of Biotechnology, Xianghu Laboratory, Hangzhou 311231, China

**Keywords:** pathotype diversity, virulence variation, genetic diversity, *Puccinia striiformis* f. sp. *tritici*

## Abstract

*Puccinia striiformis* f. sp. *tritici* (*Pst*) is adept at overcoming resistance in wheat cultivars, through variations in virulence in the western provinces of China. To apply disease management strategies, it is essential to understand the temporal and spatial dynamics of *Pst* populations. This study aimed to evaluate the virulence and molecular diversity of 84 old *Pst* isolates, in comparison to 59 newer ones. By using 19 Chinese wheat differentials, we identified 98 pathotypes, showing virulence complexity ranging from 0 to 16. Associations between 23 *Yr* gene pairs showed linkage disequilibrium and have the potential for gene pyramiding. The new *Pst* isolates had a higher number of polymorphic alleles (1.97), while the older isolates had a slightly higher number of effective alleles, Shannon’s information, and diversity. The Gansu *Pst* population had the highest diversity (*uh* = 0.35), while the Guizhou population was the least diverse. Analysis of molecular variance revealed that 94% of the observed variation occurred within *Pst* populations across the four provinces, while 6% was attributed to differences among populations. Overall, *Pst* populations displayed a higher pathotypic diversity of *H* > 2.5 and a genotypic diversity of 96%. This underscores the need to develop gene-pyramided cultivars to enhance the durability of resistance.

## 1. Introduction

Resistance to rust diseases in bread wheat cultivars (*Triticum aestivum* L., 2n = 6× = 42, AABBDD) is a common challenge due to the constant evolution of virulence in rust pathogens. *Pst* causes stripe rust (also known as yellow rust) on more than 20 grass and crop species including wheat, barley (*Hordeum vulgare* L.), triticale (×*Triticosecale* Wittmack), and rye (*Secale cereale* L.) [1]. Even though the extent of yield loss caused by stripe rust depends on factors such as pathogen aggressiveness, cultivar resistance level, and environmental variables, it has been reported that the global annual yield loss is estimated to be 1%, equivalent to USD 1 billion [2,3]. Globally, stripe rust is highly prevalent, with an occurrence rate of 88%. Moreover, the majority of wheat cultivars exhibit compatibility with this disease due to the presence of susceptible genes [3,4]. The prevalence of the pathogen is closely associated with the availability of suitable host plants and favorable weather conditions [5].

Wheat stripe rust can be managed through the utilization of cultivars carrying resistance genes, the application of fungicides, and the implementation of improved agronomic practices. Among these strategies, the deployment of resistant *Yr* genes stands out as the most effective approach due to its environmental friendliness, cost-effectiveness, and ease of implementation [6]. At present, over 80 *Yr* genes have been identified from various domesticated and wild species of wheat, comprising 54 genes conferring all-stage resistance (ASR) and 24 genes providing adult-plant resistance (APR) or high-temperature adult-plant (HTAP) resistance [7].

The diverse genetic makeup of *Pst* populations, resulting from mechanisms such as sexual recombination, mutation, and somatic hybridization, is responsible for virulence variation within the pathogen of stripe rust [8,9,10]. The wind-mediated dispersal of *Pst* urediniospores across wheat-growing environments also contributes to the genetic diversity of the pathogen [11]. Over time, these diversifications facilitate the emergence or re-emergence of highly virulent races of *Pst*, which are capable of overcoming genetic resistance in wheat cultivars. Subsequently, this results in recurrent disease epidemics [3,9,12]. For instance, the emergence of new races within the *Pst* population has led to disease outbreak, due to the breakdown of resistance for genes *Yr7*, *Yr8*, *Yr9*, *Yr10*, *Yr17*, and *Yr27* in various countries [12,13,14]. High levels of virulence have been reported in numerous characterized *Yr* genes, with the exceptions of *Yr5* and *Yr15* [7,15]. The genetic variabilities and virulence spectra of *Pst* populations have been extensively studied across countries, including Australia [11], South Africa [16], Canada [14], Russia [1], the USA [15,17,18], China [19], and Turkey [20].

From 1950 to 2000, China witnessed seven significant epidemics of stripe rust [21]. Since then, numerous research studies have been undertaken to monitor the virulence dynamics and genetic diversity of *Pst* populations. The analysis of virulence variation in *Pst* populations utilized a set of 19 differentials of wheat cultivars established in 2010, along with *Yr* single-gene differentials [7]. Chen et al. [22] identified 41 races of *Pst* from a total of 4714 isolates and they also observed changes in the virulence of CYR33 (Chinese yellow rust) on *YrSu*. Additionally, the race composition of a *Pst* population across various provinces of the country was determined [23,24]. In the hotspot provinces of China, more than 750 *Pst* races/pathotypes have been identified. These include various predominant and virulent races, namely CYR1, CYR8, CYR10, CYR13, CYR16, CYR17, CYR18, CYR19, CYR23, CYR25, CYR28, CYR29, CYR30, CYR33, and the L13 and SY11 pathotype groups [7]. The implementation of molecular markers for genotyping *Pst* populations has been widely employed in China. Using SSR markers, Chen et al. [24] identified shared multi-locus genotypes with the *Pst* populations in the provinces of Yunnan and Guizhou, which demonstrated a significant genetic resemblance between these geographical areas. Hu et al. [25] conducted a study to assess the genetic differentiation among 961 *Pst* isolates, obtained from various wheat-growing regions in the provinces of Gansu, Shaanxi, Sichuan, and Tibet. Their research findings explicitly indicated the existence of gene flow between *Pst* populations. Similarly, Yan et al. [26] confirmed genetic differentiation within individuals of isolates, while a small proportion of genetic variation was observed between the *Pst* populations of Yunnan and the Yangtze river. Furthermore, the simple nucleotide polymorphism (SNP) and amplified fragment length polymorphism (AFLP) techniques are commonly utilized in China for genotyping *Pst* populations [27,28]. In China, the provinces of Gansu, Sichuan, Guizhou, and Yunnan have been identified as epidemiological zones, exhibiting frequent variations in the virulence of stripe rust [21].

Understanding the temporal and spatial dynamics of *Pst* populations is essential for the implementation of timely and effective disease management strategies in these high-risk regions. In this study, we characterized and compared the virulence patterns of two groups of *Pst* isolates collected during the epidemic seasons of 2016 and 2023. The specific objectives of this study were to (1) analyze the pathotypes of *Pst* collections based on the Chinese differentials, (2) determine the frequency of virulence and effective genes using *Yr* single-gene differentials, and (3) assess the genetic diversity among *Pst* populations using molecular markers.

## 2. Materials and Methods

### 2.1. Collection of Pst Isolates

Wheat leaves exhibiting symptoms of wheat stripe rust were systematically collected from experimental plots, nurseries, and commercial fields located across the provinces of Yunnan, Guizhou, Sichuan, and Gansu. To obtain an adequate quantity of urediniospores and a representative sample of isolates, a total of five infected leaves exhibiting *Pst* uredinia from a single cultivar or field were collected and considered as a single sample. Two rounds of field surveys were conducted during the cropping seasons of 2023 and 2016. For the comparative analysis of virulence and genotypic variation, isolates collected in the 2016 cropping season were designated as old isolates of Yunnan (YNOI), Guizhou (GZOI), Sichuan (SCOI), and Gansu (GSOI), while those collected in the 2023 cropping season were designated new isolates of Yunnan (YNNI), Guizhou (GZNI), and Sichuan (SCNI). Leaf samples were dried with moisture absorbent for 24 h and stored at 4 °C until further use.

### 2.2. Isolate Identification and Multiplication

To obtain fresh urediniospores, 3–5 cm segments of infected leaves from each sample were placed on wet absorbent paper in Petri dishes and were incubated in a dark room at 10 °C for 24 h [29,30]. Fresh urediniospores produced by a single uredinium were inoculated onto 10-day-old susceptible seedlings of Mingxian 169 (MX169), using a sterilized needle for isolate multiplication. The MX169 seedlings were treated with a 0.33% maleic hydrazide solution to inhibit further growth [31]. Urediniospores were harvested 14–18 days after inoculation and were dried in a desiccator at 5 °C for 24–48 h. Urediniospores produced from a single uredinium on each leaf were considered as a single isolate. In total, 143 isolates of *Pst* were recovered from 160 samples. *Pst* isolates were temporarily stored at 4 °C, or at −40 °C, for months. Long-term stored isolates were heat-shocked at 42 °C for 5 min to reactivate and were then stored at 4 °C for 12 h.

### 2.3. Inoculation of Chinese Differentials and Yr Single Gene Lines

Nineteen Chinese differentials and 31 single *Yr* gene differentials were planted in pots (10 × 10 × 7 cm^3^) and boxes (10 × 25 × 50 cm^3^), respectively (Supplementary Table S1). MX169 was included as the susceptible check. Growth conditions and inoculation procedures were previously described [29]. Seedlings were grown in a growth chamber, programmed at 15 ± 1 °C/12 ± 1 °C with a photoperiod of 16 h light/8 h dark, maintaining a relative humidity of 70%. Seedlings at the two-leaf stage were sprayed with 15–20 mg of urediniospores suspended in 1 mL of Novec 7100 engineered fluid (3M, Maplewood, MN, USA). Inoculated seedlings were then placed in a dark dew chamber at 10 °C for 24 h, before being transferred to growth chambers automated with a diurnal temperature cycle ranging from 4 °C at 2:00 AM to 20 °C at 2:00 PM, 8 h dark and 16 h light from 10 PM to 6 AM and from 6 AM to 10 PM, respectively. Stringent measures were taken to prevent cross-contamination during the inoculation, incubation, and handling of isolates.

### 2.4. Characterization of Virulence

The infection types (ITs) produced by *Pst* isolates on seedlings of Chinese differentials and *Yr* single-gene lines were assessed after complete sporulation of MX169, using a previously described 0–4 scale [22], where 0 = absence of visible symptoms; 0; = presence of necrotic and/or chlorotic flecks, without sporulation; 1 = presence of necrotic and/or chlorotic blotches or stripes, with trace sporulation; 2 = presence of necrotic and/or chlorotic blotches or stripes, with moderate sporulation; 3 = presence of chlorotic blotches or stripes, with moderate to abundant sporulation; and 4 = absence of necrosis or chlorosis, with abundant sporulation. ITs 0–2 were considered as pathogen avirulence, while ITs 3–4 were deemed as pathogen virulence. Isolates lacking clear virulence patterns on the testing materials were subjected to re-inoculation to verify their virulence patterns.

### 2.5. Pathotype Designation and Effectiveness of Yr Genes

*Pst* pathotypes were identified and characterized based on the virulence formulae of the 19 Chinese differentials, using the methodology outlined by Draz [32]. For instance, pathotype *V2*, *4*, *8*, *14* exhibited virulence on the corresponding differentials of Fulhard (unknown), Mentana (unknown), Funo (*YrA+*), and Suwon 11 (*YrSu*). *Pst* isolates displaying similar virulence patterns on the differentials were grouped together as a single pathotype, while those displaying unique virulence patterns were classified as distinct pathotypes. The efficacy levels of the *Yr* genes were classified as high, moderate, or low, based on their respective virulence frequencies of 0–20%, 20–50%, and 50–100%, as previously described [15].

### 2.6. Association of Yr Genes

Pairwise virulence/avirulence associations of *Yr* loci were empirically assessed according to Parks et al. [33]. Four pairwise groups of alleles at two loci were identified, as follows: isolates virulent to both *Yr* genes (VV); isolates avirulent to both *Yr* genes (AA); isolates virulent to one *Yr* gene, but avirulent to the other (VA); and isolates avirulent to one *Yr* gene, but virulent to the other (AV). Positive associations were determined if the AA or VV groups represented more than 60% of the tested isolates, while negative associations were determined if AV or VA accounted for more than 60% of the isolates. If the combined frequency of AA and VV fell within the range of 40–60%, the association was deemed unclear.

### 2.7. Molecular Marker Analysis

DNA was extracted from 20 mg of dried urediniospores from each isolate, using the cetyl trimethyl ammonium bromide method [34]. Prior to PCR amplification, the quality and quantity of the extracted DNA were determined using an ND-1000 spectrophotometer (Thermo Scientific, Waltham, MA, USA), followed by dilution with ddH_2_O to a concentration of 50 ng/μL. The genotyping of DNA samples was conducted using eleven previously designed SSR markers including RJ3 and RJ22 [35]; RJ3N and RJ13N [36]; CPS08, CPS09, CPS13, and CPS27 [37]; and PstP002, PstP003, and PstP006 [38]. These markers were chosen for their high informativeness. Primers of these markers were synthesized by Sangon Biotech Inc. (Shanghai, China, http://www.sangon.com/, accessed on 10 February 2024).

A reaction mixture consisted of 25 μL, comprising 2 μL of DNA (50 ng/μL), 2.5 μL of 10× reaction buffer (Mg^2+^-free), 2 μL of Mg^2+^ (25 mM), 2 μL of dNTPs (2.5 mM), 1 μL of each primer (10 mM), 0.2 μL of *Taq* DNA polymerase (5 U/μL), and 14.3 μL of ddH_2_O. The amplification of DNA was carried out in a thermocycler (Bio-Rad, Los Angelas, CA, USA) under the following conditions: initial denaturation at 94 °C for 5 min, 30 cycles of denaturation at 94 °C for 30 s, annealing at 50–58 °C for 30 s, extension at 72 °C for 1 min, and a final extension at 72 °C for 10 min. The amplified products were resolved using capillary electrophoresis technology [39].

### 2.8. Data Analysis

The diversity of pathotypes in the *Pst* population was assessed using the Shannon–Wiener diversity index (*H*) [40]. According to this index, low, moderate, and high diversities were indicated with values of *H* < 1.5, 1.5 < *H* < 2.5, and *H* > 2.5, respectively.

The data scored using a 0–4 scale were transformed into a coding system, where a value of 1 represents ratings of 3–4, indicating virulence, while a value of 0 represents ratings of 0–2, indicating avirulence. The virulence frequency (VF) indicates the percentage of virulent isolates of *Pst* in a population to an individual *Yr* gene line. The virulence complexity (VC) of each isolate indicates the isolate’s potential to overcome host *Yr* genes. VF and VC were analyzed using a Jaccard dissimilarity coefficient in the virulence analysis tool (VAT) [41]. The statistical significance of virulence frequencies among subpopulations of *Pst* was assessed using the *t*-test (https://www.scribbr.com/statistics/t-test/, accessed on 2 March 2024). The unique virulence patterns (UVPs), average virulence complexity (AVC), relative virulence complexity (RVC), and virulence uniformity (VU) of the *Pst* isolates were also computed using VAT [41].

A dendrogram showing the virulence patterns of both old and new *Pst* isolates was generated using the unweighted pair group method with arithmetic mean (UPGMA), in the SAHN program of NTSYSpc 2.2 [42]. Genetic diversity parameters, including number of alleles (*Na*), number of effective alleles (*Ne*), Shannon index (*I*), unbiased diversity (*uh*), percentage of polymorphic loci (*PPL*), and analysis of molecular variance (AMOVA) with 999 permutations, were analyzed using GenAlEx 6.5 [43]. The Nei’s genetic distance and principal coordinate analysis (PCoA) plots were generated using GenAlEx 6.5 [43].

## 3. Results

### 3.1. Identification of Pathotypes and Diversities in the New and Old Isolates of Pst

The analysis of virulence patterns among 143 *Pst* isolates on the 19 Chinese wheat differentials for stripe rust revealed 98 distinct pathotypes (Supplementary Table S2). Nineteen *Pst* pathotypes were detected more than once, with virulence frequencies ranging from 1.4% to 5.0% (Table 1). The most frequent pathotype was *V2*, *4*, *8*, *14*, with a pathotype frequency of 5.0% (7 out of 143) in the Guizhou, Sichuan, and Yunnan provinces. This pathotype exhibited virulence on the differential lines Fulhard, Mentana, Funo, and Suwon 11, and was avirulent on all other differentials including Trigo Eureka, Lutescens 128, Virgilio, Abbondanza, Early Premium, Danish 1, Jubilejina 2, Fengchan 3, Lovrin 13, Kangyin 655, Zhong 4, Lovrin 10, Hybrid 46, *T. spelta* var *album*, and Guinong 22. The second most frequent pathotype, *V2*, *4*, *8*, *12*, *14*, *16*, had a frequency of 4.2% in the Sichuan and Gansu *Pst* populations. Pathotypes *V2*, *8*, *14*, and *V0* were each detected five times (3.5%). The former was found in both new and old isolate groups of Yunnan, Guizhou, and Sichuan, while the latter was detected in the old isolate groups of the Yunnan, Guizhou, and Gansu provinces. Three pathotypes (*V2*, *14*; *V2*, *8*, *14*, *19*; and *V2*, *8*) each had frequencies of 2.8% across the old isolates of the provinces. Pathotypes *V2*, *7*, *8*, *14*, *19*; *V2*, *8*, *19*; and *V2* were detected, each at 1.4%, across old isolates of the provinces. Pathotypes *V4*, *14*, *16* and *V2*, *4*, *14*, 16 had virulence frequencies of 2.8% and 2.1% in the new isolate groups of the Guizhou and Sichuan provinces, respectively. Similarly, *V2*, *3*, *4*, *6*, *7*, *8*, *10*, *11*, *12*, *14*, *16*, *19*; *V2 10*; *V2*, *4*, *8*, *11*, *14*, *16*; and *V2*, *8*, *14*, *16* were detected two times (1.4%) each across new isolates of the provinces. Six pathotypes (*V2*, *4*, *8*, *14*; *V2*, *8*, *14*; *V2*, *4*, *8*, *10*, *14*, 19; V3, *7*, *10*, *19*; *V2*, *4*, *8*, *12*, *14*, *16*; and *V2*, *4*, *7*, *8*, *14*) with frequencies ranging from 1.4% to 5.0% were detected in both isolate groups. The remaining 79 pathotypes were each detected only once (Supplementary Table S2).

Pathotype diversity was comparable in Yunnan and Gansu (each with *H* = 3.6), as well as in Sichuan (*H* = 3.5). However, pathotype diversity was lower in the Guizhou *Ps*t population, with an *H* value of 2.8 (Table 1). These data provide strong evidence for the higher diversity and uniform distribution of pathotypes across the *Pst* populations.

### 3.2. Virulence Complexities and Frequencies on the Chinese Differentials

**Figure 1:**
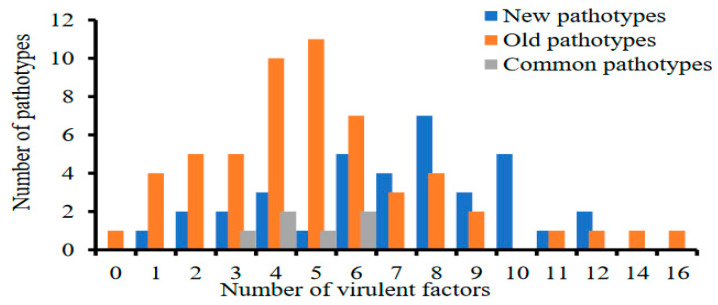
Distributions of *Pst* pathotypes according to their virulence complexity.

The virulence complexities (VCs) of old pathotypes (derived from old isolates) ranged from 0 to 16, with a mean of 5.2 (Figure 1). Pathotype *V0* displayed avirulence to all differential sets. Conversely, pathotype *V1*, *2*, *3*, *4*, *5*, *6*, *7*, *8*, *9*, *10*, *11*, *12*, *13*, *14*, *16*, *17* was virulent to all differentials, except Zhong 4, *T. spelta* var *album*, and Guinong 22. In new pathotypes (derived from new isolates), the VCs ranged from 1 to 12, with a mean of 7. Pathotype *V12* was solely virulent to Lovrin 13, while pathotype *V2*, *3*, *4*, *5*, *7*, *8*, *9*, *10*, *11*, *12*, *14*, *16* showed virulence to Fulhard, Lutescens 128, Mentana, Virgilio, Early Premium, Funo, Danish 1, Jubilejina 2, Fengchan 3, Lovrin 13, Suwon 11, and Lovrin 10. In both pathotype groups, narrow virulence (e.g., 0–1) and wide virulence (e.g., 11–16) were limited. Instead, virulence between 2 and 10 differentials tended to be frequently observed among the *Pst* pathotypes (Supplementary Table S2 and Figure 1).

**Figure 2:**
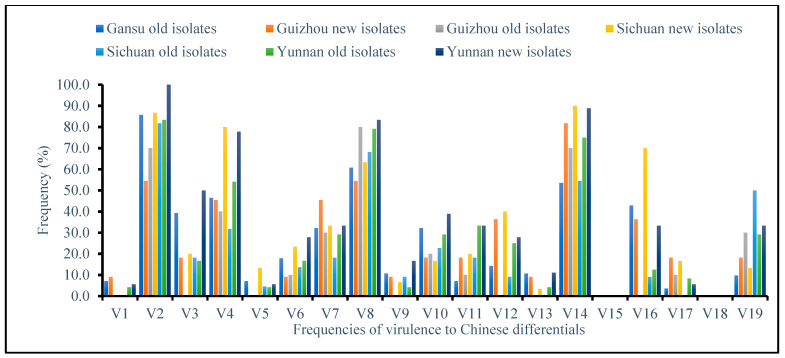
Frequencies of virulence in both new and old *Pst* isolates on 19 Chinese differentials across provinces.

Virulence frequencies in the new isolates were higher, ranging from 56 to 100% for Fulhard, 50 to 80% for Mentana, 56 to 83% for Funo, and 82 to 90% for Suwon11, compared to 78–86%, 40–54%, 60–80%, and 54–71% in the old isolates, respectively (Figure 2). Virulence to Lutescens 128 and Lovrin 10 was as high as 70% and 50% in the new isolates, respectively, while it was as low as 39% and 43% in the old isolates. Virulence was moderate, though higher in the new isolates for Abbondanza (9–28%), Early Premium (33–46%), Jubilejina 2 (17–39%), Lovrin 13 (28–44%), and Hybrid 46 (6–20%), compared to 9–18%, 18–32%, 20–32%, 9–28%, and 4–10% in the old isolates, respectively. Only Guinong 22 exhibited a higher virulence (50%) to the old isolates than to the new ones (33%). Virulence to Trigo Eureka, Virgilio, Danish 1, Fengchan 3, and Kangyin 655 was less than 20% across isolate groups. Neither isolate group exhibited virulence to Zhong 4 and *T. spelta* var *album*.

### 3.3. Virulence Frequencies and Complexities on Yr Single-Gene Differentials

The frequencies of virulence to 31 *Yr* single-gene differentials ranged from 0 to 93% (Table 2). All *Pst* isolates were avirulent to *Yr5* and *Yr15*. Low frequencies of virulence were observed for *Yr24* (10–20%) and *Yr26* (0–20%); moderate frequencies to *Yr76* (18–50%), *Yr50* (0–42%), *Yr41* (0–35%), *Yr32* (0–47%), *Yr10* (0–41%), *Yr17* (13–50%), *YrSp* (0–40%), and *YrTr1* (0–32%); and high frequencies to *Yr45* (63–92%), *YrSu* (58–82%), *Yr43* (57–75%), *Yr25* (50–90%), *YrJu4* (55–88%), and *YrRes* (58–93%) across the provinces. Some *Yr* genes showed province-specific effectiveness. Frequency of virulence to *Yr21* exceeded 55% in the Yunnan, Guizhou, and Gansu *Pst* populations, but remained below 37% in Sichuan. Virulence to *Yr1* was less than 30% in Guizhou, but ranged from 30% to 55% in the other provinces. Virulence to *Yr40* was less than 40% in the Guizhou and Gansu provinces, whereas it reached as high as 72% in Sichuan and 68% in Yunnan. Virulence to *Yr64* was 80% in Guizhou, but below 50% in the other provinces.

*Yr* genes, such as *Yr50*, *Yr41*, *Yr10*, *Yr17*, *YrSp*, *YrTr1*, *Yr26*, and *Yr29*, exhibited significantly higher frequencies of virulence to the new isolates than the old ones (*p* < 0.05). Virulence to *Yr50* ranged between 10% and 42% in the new isolates, while an immune response was observed in the old isolates. Virulence to *Yr41*, *Yr10*, *Yr17*, *YrSp*, *YrTr1*, *Yr26*, and *Yr29* was between 0 and 21%, 0 and 11%, 13 and 25%, 0 and 25%, 0 and 8%, 0 and 11%, and 30 and 55% in the old isolates. However, it increased in the new isolates, with the virulence to the corresponding genes of 30–35%, 10–41%, 26–50%, 21–40%, 20–32%, 17–20%, and 55–79%, respectively. Virulence to *Yr76*, *Yr40*, *YrA*, *Yr1*, *Yr6*, *Yr3c*, *Yr7*, *Yr9*, *Yr32*, *Yr64*, and *YrRes* was also substantially increased in the new isolates, despite being statistically non-significant. In contrast, *Yr45*, *Yr25*, *Yr8*, *Yr44*, and *YrKy2* exhibited higher frequencies of virulence in the old isolates of *Pst*.

Each *Pst* population displayed 100% UVP (Table 3). New isolates from the Guizhou and Sichuan provinces exhibited the highest AVC, rendering each 14.4 out of 31 *Yr* genes ineffective. The AVC was comparable, with values of 13.6 and 13.7 observed in old isolates from Gansu and new isolates from Yunnan, respectively. AVC values of 11.5 and 12.4 were noted in the old isolates from the Guizhou and Yunnan provinces, respectively. The AVC decreased to 10.6 in the old isolates from Sichuan. A similar trend between AVC and RVC was noted among the *Pst* populations. The highest VU (0.36) was computed from Sichuan’s old isolates. Guizhou’s new isolate and Gansu’s old isolates exhibited the lowest VU values of 0.23 and 0.24, respectively.

### 3.4. Associations of Alleles at the Avirulence Loci

The pairwise association between virulence and avirulence of *Pst* isolates at *Yr* loci are presented in Table 4. Twenty-three *Yr* gene pairs showed a positive association. However, the association between *Yr50* and *YrJu4*, *Yr50* and *YrKy2*, and *Yr41* and *YrKy2* remained unclear, as the proportion of AA plus VV fell within the range of 40–60%, despite statistical significance.

### 3.5. Cluster Analysis of Virulence

**Figure 3:**
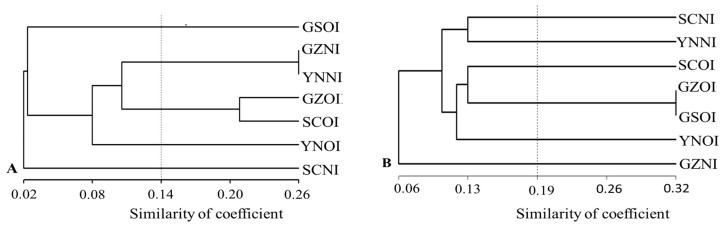
Dendrograms showing virulence similarity among *Pst* isolates defined by provinces and isolate types, using the 19 Chinese differentials (**A**) and the 31 single *Yr* gene lines (**B**).

Cluster analysis was performed based on the virulence data of the *Pst* populations using two sets of differentials (Figure 3). At a similarity coefficient of 0.14, the *Pst* populations were grouped into five clusters, based on the 19 sets of Chinese differentials (Figure 3A). New isolates from the Guizhou and Yunnan provinces, as well as old isolates from the Guizhou and Sichuan provinces, were clustered separately. Old isolates from Yunnan and Gansu, as well as new isolates from Sichuan were clustered independently. Old isolates from Gansu and new isolates from Sichuan are the most distantly clustered isolates. Additionally, based on the virulence frequencies of 31 single *Yr* genes, the *Pst* populations were further grouped into six clusters (Figure 3B). With the exception of the old isolates of the Guizhou and Gansu provinces, the remaining populations were independently clustered.

### 3.6. Molecular Diversity

The diversity parameters of the *Pst* populations, as determined using SSR markers, are presented in Table 5. The number of alleles (*Na*) was higher in the new isolates (*Na =* 1.97) compared to the old isolates (*Na =* 1.94). On the other hand, the number of effective alleles (*Ne =* 1.58), Shannon’s information index (*I =* 0.51), and unbiased diversity (*uh =* 0.34) were higher in the old isolates compared to the new ones. A provincial comparison revealed that the highest *Na* value (2.00) was observed in the Sichuan and Yunnan populations. The maximum *Ne* (1.56), *I* (0.51), and *uh* (0.35) values were observed in the Gansu population, followed by the Yunnan population with *Ne*, *I*, and *uh* values of 1.56, 0.51, and 0.34, respectively. In the Sichuan population, these values were 1.54, 0.49, and 0.33, respectively. The Guizhou population exhibited the lowest values of genetic parameters *Na* (1.81), *Ne* (1.54), *I* (0.46), *uh* (0.32), and percentage of polymorphic loci (86%). The average polymorphism value among the *Pst* subpopulations was 96%.

The AMOVA was used to partition the molecular variance among four provinces of the *Pst* populations (Yunnan, Guizhou, Sichuan, and Gansu), as well as between the old and new isolate groups. The results showed that 94% of the genetic variance existed within populations, while 6% was among the *Pst* populations in the four provinces. Within the isolate groups, we observed 89% of genetic variance, whereas 11% of the genetic variance was attributed to the differences between the old and new isolates of *Pst* (*p* = 0.001) (Table 6).

### 3.7. Genetic Distance

**Figure 4:**
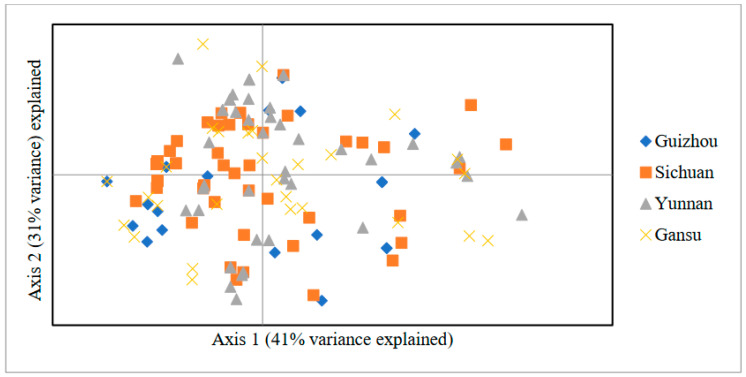
Principal coordinates analysis (PCoA) showing four *Pst* populations collected from the Yunnan, Guizhou, Sichuan, and Gansu provinces.

The genetic distance among the *Pst* populations in the provinces of Yunnan (YN), Guizhou (GZ), Sichuan (SC), and Gansu (GS) are presented in Table 7 and Figure 4. The Nei’s distance ranged from 0.03 to 0.10. There was a decrease in genetic distance between the populations of GS-SC, GS-YN, and SC-YN, implying that the SC-YN populations were genetically closer to each other than the other populations. The genetic distance was comparable between population pairs SC-GZ, YN-GZ, and GS-GZ, each with a value of 0.06. However, the populations of GS-SC were found to be the most genetically distant from the others. The results are consistent with the cluster analysis of virulence (Figure 3).

A principal coordinate analysis (PCoA) plot was generated based on the Nei’s distances computed between every pair of SSR genotypes. The majority of populations did not show distinct separation, except for the Guizhou population, which was predominantly clustered within the third and the fourth quadrants (Figure 4). PC1 (axis1) and PC2 (axis 2) accounted for 41% and 31% of the total genetic variance, respectively.

## 4. Discussion

The frequent outbreaks of wheat stripe rust in China led to the constant replacements of cultivars [21,44]. The northwestern (including Gansu) and the southwestern regions (including Yunnan, Guizhou, and Sichuan) of China are among the largest over-summering and over-wintering regions of *Pst* [45]. Monitoring the phenotypic and genetic diversity of *Pst* in these regions is essential to understand the pathogen’s population structure and to identify effective *Yr* genes for breeding programs. The virulence profiles of the *Pst* isolates on the Chinese differentials revealed a wide distribution of pathotypes across provinces. Approximately 19% of pathotypes (44% of isolates) were detected more than once within or among provinces. This phenomenon is likely attributed to the migration of spores between provinces. *Pst* pathotypes, including *V2*, *8*, *14*; *V3*, *7*, *10*, *19*; *V2*, *4*, *8*, *14*; and *V2*, *4*, *8*, *10*, *14*, *19*, were consistently found in both old and new isolates. The presence of ancestral isolates in the current populations of *Pst* indicates their capacity to adapt to diverse environmental factors in the past eight years. In contrast, pathotypes such as *V0*; *V2*, *8*; *V2*, *8*, *19*; and *V2*, *8*, *14*, *19* were prevalent in old isolates, but were not detected in new ones, indicating a potential genetic drift in these specific pathotypes. This result is consistent with the observation of the previous report [21].

It was observed that the newly identified isolates exhibited a higher frequency of virulence to the Chinese and *Yr* single-gene differentials compared to the old isolates, thereby suggesting a shift in the virulence. Two possible explanations can be derived. First, mutations, as a mechanism of virulence variation, give rise to new isolates through evolution [19]. In most cases, mutations increase the magnitude of virulence [46]. Second, the newly emerged isolates of *Pst* were found to be more aggressive than the old isolates [17]. However, the high virulence uniformity observed in the old isolates from Sichuan province should not be underestimated. This uniformity in virulence could increase the risk of stripe rust outbreaks, since the virulence profiles on *Yr* genes remain consistent. It was observed that the *Pst* populations collected from different provinces during the 2016 or 2023 epidemic seasons demonstrate a higher virulence similarity within their respective populations. Conversely, a lower degree of virulence similarity was observed between the *Pst* populations collected during the two epidemic seasons. This suggests that the changes introduced between 2016 and 2023 have a greater impact on *Pst* virulence dynamics compared to the environmental variations among the provinces at a given time. Previous studies have revealed that the populations of *Pst* from Yunnan and Guizhou are closely related, while those from Sichuan and Gansu exhibit distinct clustering patterns [47], which is consistent with our findings. Similarly, in Europe, *Pst* isolates collected before 2011 from the United Kingdom and France showed minimal genetic diversity. Nevertheless, these isolates were genetically distinct from those collected in 2013 [48].

With the exception of trace sporulation (IT 1) on a 0–4 scale [22], incompatibility was observed between the *Pst* isolates and Zhong 4 (unknown gene), *Yr5*, and *Yr15*. This suggests their effectiveness against *Pst* isolates. The effectiveness of Zhong 4, *Yr5*, and *Yr15* has been extensively documented [15,29,44,49]. However, instances of virulence to *Yr15* in Afghanistan [50] and *Yr5* in India, Australia, Tajikistan [11], Turkey [51], and Syria [52] have been reported. In China, virulence on *Yr5* was detected in the Shaanxi and Qinghai provinces [53]. Due to their close geographical proximity, the incursion of *Yr5* virulent isolates through wind from Shaanxi and Qinghai to Sichuan, Guizhou, and Gansu provinces is likely to occur. Thus, continuously monitoring the *Pst* population structures is a crucial task. Virulence to *Yr76*, *Yr50*, *Yr32*, *Yr24, Yr10*, *Yr17*, *YrSp*, *YrTr1*, and *Yr26* was reached at a moderate level in the studied provinces. The moderate virulence signals a potential risk of complete resistance loss under high selection pressure. Other *Yr* genes displayed a province-specific effectiveness. For instance, *Yr6* and *Yr21* exhibited a moderate level of effectiveness against the *Pst* population in the Sichuan province. However, their resistance was overcome by the isolates found in the remaining provinces. *Yr40* was effective against the Guizhou isolates, but ineffective to the Sichuan and Yunnan isolates. This location-specific efficacy suggests the limited applicability of resistance genes across provinces. High selection pressure on *Yr45*, *YrSu*, *Yr43*, *YrA*, *Yr25*, *Yr7*, *Yr9*, *Yr29*, *YrJu4*, and *YrRes* was observed across the *Pst* isolate groups. Among them, *Yr7*, *Yr9*, *Yr29*, *YrSu*, and *YrA* have been deployed widely in Chinese wheat cultivars [54]. However, the continuous cultivation of these susceptible cultivars may lead to an accumulation of inoculum, thereby increasing disease pressure over time. Therefore, it is essential to replace these cultivars at the regional level with alternative cultivars that possess *Yr5* and *Yr15*. Compared to the 2016 survey conducted by Chen et al. [55], dynamics in virulence were observed among *Yr* genes. Consistency with our results was noted for virulence to *Yr5*, *Yr7*, *Yr43*, and *Yr44*. While increases in virulence were observed for *Yr8*, *Yr17*, *Yr24*, *Yr32*, and *YrTr1*, *Yr6*, *Yr9*, *YrSp*, and *Yr76* exhibited a clear decline in virulence. Likewise, our results showed a relatively lower frequency of virulence compared to previous studies in the Yunnan and Guizhou provinces [24]. This dynamic in frequency of virulence could be attributed to factors such as mutation, selection, sexual recombination, and migration [56].

Associations among alleles at pairs of avirulence loci enhance the durability of resistance against stripe rust. Several *Yr* gene pairs exhibited positive association, with a VV proportion of less than 10%. For instance, there was a significant association between avirulence to *Yr24* and *Yr26* in the *Pst* isolates. These *Yr* genes have been frequently used as sources of resistance genes in wheat breeding programs in China [57]. However, the emergence of virulent races to *Yr24* and *Yr26* has been reported in the Sichuan, Gansu, Shaanxi, Ningxia, and Qinghai provinces, posing a threat to wheat production [55,58]. This underscores an urgent need for pyramiding *Yr24* and *Yr26*, as the co-selection likelihood of these *Yr* genes by the pathogen is low. Similarly, 22 *Yr* gene pairs were found to be positively associated with a lower proportion of virulence compared to their individual virulence frequency. This demonstrates that pyramiding these genes could serve as a viable strategy to extend the duration of resistant cultivars in production.

Both the phenotypic and genotypic results revealed the presence of genetic diversity in the *Pst* population. The comparison among provinces showed that the *Pst* population of Gansu exhibit a higher genetic diversity. This can be attributed to favorable environmental conditions that support over-summering and over-wintering, along with the presence of alternative hosts like *Berberis* spp., which encourage sexual recombination within the population [59]. Previous studies have confirmed the existence of genotypic diversity within the *Pst* population in Gansu province [27,60,61]. Moreover, the Yunnan *Pst* population exhibited a high level of pathotypic and genotypic diversity. The cultivation of various wheat genotypes throughout the year in Yunnan likely increases the genetic diversity of *Pst* [61]. The cultivation of various wheat cultivars frequently leads to the deployment of diverse *Yr* genes, thereby augmenting the overall diversity of the pathogen population. A diverse pathogen population increases the likelihood of the emergence of more virulent pathotypes through mechanisms such as mutation, recombination, and host selection [8,9,10]. Consequently, this can lead to the occurrence of novel disease epidemics. To mitigate this risk, regions with high pathogen diversity should implement a broader range of breeding programs, aiming to develop resistant cultivars that can effectively combat the disease. On the contrary, the *Pst* isolates from Guizhou province exhibited the lowest diversity, presumably due to unfavorable weather conditions, narrow host range, geographic barriers, and a small sample size.

The results of AMOVA revealed that the majority of genetic differentiation occurred within populations, despite limited genetic variation observed among populations. This is consistent with a previous study [25]. Several factors influence the level of genetic differentiation in *Pst* populations. Gene flow increases genetic variation within a population, while decreasing genetic differentiation between populations [25]. The lack of distinct clustering among *Pst* populations in the PCoA signifies the likelihood of gene flow among provinces. It is well-known that mutation and somatic recombination can augment genetic variation within populations [9]. Similarly, studies have confirmed that sexual recombination significantly increases the degree of genetic variability within *Pst* populations [8,25]. However, natural selection and genetic drift contribute to genetic differentiation between populations [19]. This suggests that the collective effect of these factors could determine the genetic variation among *Pst* populations.

Despite limited genetic variation observed among provinces, the *Pst* populations of Gansu and Sichuan were found to be genetically distant. The urediniospores of *Pst* exhibit a migratory behavior towards the mountainous regions of Gansu during the summer season, to undergo over-summering in favorable conditions. Similarly, during the winter season, these spores move to the lower-elevation areas of Sichuan for over-wintering, taking advantage of the cold conditions prevalent in that region [62]. Gradually, the *Pst* populations could potentially limit or even stop such migratory behavior, undergoing location-specific evolutionary changes to cope with temperature fluctuations. Consequently, this could result in a divergence in their genetic makeup. Recent evidence has confirmed distinct clustering patterns among *Pst* isolates from Sichuan and Gansu [47].

In conclusion, it can be deduced that the newly evolved isolates of *Pst* demonstrate a higher virulence complexity, leading to the ineffectiveness of 14.2 out of 31 *Yr* genes, compared to older isolates, which rendered only 12 *Yr* genes ineffective. All *Pst* isolates were avirulent to lines with *Yr5*, *Yr15*, and Zhong 4. These resistant *Yr* genes can be recommended for stripe rust breeding programs in China. The *Pst* population exhibited higher pathotypic and genotypic diversities across the studied provinces of China. These findings underscore the necessity to develop multiple resistant cultivars or employing gene-pyramiding strategies to mitigate the risks associated with the diversity of the pathogen.

## Figures and Tables

**Table 1:** Distribution and diversity of *Pst* pathotypes across the four provinces in China.

No.	Pathotype ^a^	Yunnan	Guizhou	Sichuan	Gansu	No. Isolates/Mean	Frequency (%)
1	*V2*, *4*, *8*, *14*	(2)	1 (2) ^b^	1 (1)		7	5.0
2	*V2*, *4*, *8*, *12*, *14*, *16*			5	(1)	6	4.2
3	*V2*, *8*, *14*	1 (1)	1	1 (1)		5	3.5
5	*V0*	(1)	(2)		(2)	5	3.5
4	*V4*, *14*, *16*		1	3		4	2.8
6	*V2*, *14*	(2)	(1)	(1)		4	2.8
7	*V2*, *8*, *14*, *19*	(1)	(1)	(2)		4	2.8
8	*V2*, *4*, *8*, *10*, *14*, *19*	2	(1)	(1)		4	2.8
9	*V2*, *8*			(2)	(2)	4	2.8
10	*V2*, *4*, *14*, *16*			3		3	2.1
11	*V2*, *3*, *4*, *6*, *7*, *8*, *10*, *11*, *12*, *14*, *16*, *19*	1		1		2	1.4
12	*V2*, *10*	1		1		2	1.4
13	*V3*, *7*, *10*, *19*		1	(1)		2	1.4
14	*V2*, *4*, *8*, *11*, *14*, *16*	1	1			2	1.4
15	*V2*, *8*, *14*, *16*	2				2	1.4
16	*V2*, *8*, *19*			(2)		2	1.4
17	*V2*, *7*, *8*, *14*, *19*	(1)			(1)	2	1.4
18	*V2*, *4*, *7*, *8*, *14*	(1)	1			2	1.4
19	*V2*		(1)		(1)	2	1.4
	*H* ^c^	3.6	2.8	3.5	3.6	3.4	

^a^ Pathotypes detected more than once are listed with their corresponding virulence (*V1–V19*) to the 19 Chinese differentials: *V0 =* avirulent to all differentials; *V1 =* Trigo Eureka (*Yr6*); *V2 =* Fulhard (unknown); *V3 =* Lutescens 128 (unknown); *V4 =* Mentana (unknown); *V5 =* Virgilio (*YrVir1* and *YrVir2*); *V6 =* Abbondanza (unknown); *V7 =* Early Premium (unknown); *V8 =* Funo (*YrA*,*+*); *V9 =* Danish 1 (*Yr3*); *V10 =* Jubilejina 2 (*YrJu1*, *YrJu2*, *YrJu3*, and *YrJu4*); *V11 =* Fengchan 3 (*Yr1*); *V12 =* Lovrin 13 (*Yr9*,*+*); *V13 =* Kangyin 655 (*Yr1, YrKy1*, and *YrKy2*); *V14 =* Suwon 11 (*YrSu*); *V15 =* Zhong 4 (unknown); *V16 =* Lovrin 10 (*Yr9*); *V17 =* Hybrid 46 (*Yr3b* and *Yr4b*); *V18* = *T. spelta* var *album* (*Yr5*); and *V19 =* Guinong 22 (*Yr26*). ^b^ Old and new isolates are shown inside and outside parentheses, respectively. ^c^ Shannon–Wiener pathotype diversity index (*H*) for the 143 *Pst* isolates.

**Table 2:** Virulence frequencies of 143 *Pst* isolates on 31 *Yr* genes of wheat lines at the seedling stage.

*Yr* Genes	New Isolates				Old Isolates					*p*-Value
	SCNI (29)	YNNI (19)	GZNI (11)	Mean	SCOI (22)	YNOI (24)	GZOI (10)	GSOI (28)	Mean	
*Yr21*	24.1	57.9	64.0	48.7	36.4	55.2	60.0	57.1	51.9	0.400
*Yr76*	31.0	47.4	50.0	42.8	18.2	20.8	44.4	22.2	26.4	0.059
*Yr50*	37.9	42.1	10.0	30.0	0.00	0.00	0.00	0.00	0.00	0.008
*Yr40*	72.4	68.4	30.0	56.9	36.4	50.0	40.0	32.2	39.7	0.107
*Yr41*	34.5	31.6	30.0	32.0	13.6	4.20	0.00	21.4	9.80	0.006
*Yr45*	69.0	63.2	91.0	74.4	81.8	91.7	90.0	82.1	86.4	0.090
*YrSu*	72.4	57.9	82.0	70.8	63.6	58.3	70.0	75.9	67.0	0.314
*Yr43*	62.1	57.9	73.0	64.3	72.7	75.0	70.0	57.1	68.7	0.251
*YrA*	58.6	52.6	46.0	52.4	31.8	54.2	50.0	60.7	49.2	0.351
*Yr25*	58.6	50.4	82.0	63.7	63.6	83.3	90.0	75.0	78.0	0.114
*Yr1*	55.2	47.4	30.0	44.2	31.8	30.8	20.0	46.4	31.8	0.265
*Yr5*	0.00	0.00	0.00	0.0	0.00	0.00	0.00	0.00	0.00	
*Yr6*	45.2	68.4	46.0	56.5	18.2	54.2	60.0	53.6	46.5	0.230
*Yr3c*	44.8	57.9	60.0	54.2	31.8	41.7	60.0	46.4	45.0	0.150
*Yr7*	82.8	55.6	91.0	76.5	45.5	58.3	70.0	71.4	61.3	0.121
*Yr8*	27.6	47.4	30.0	35.0	59.1	62.5	70.0	64.3	64.0	0.002
*Yr9*	58.6	52.6	60.0	57.1	27.3	62.5	30.0	57.1	44.2	0.146
*Yr24*	20.0	20.0	10.0	16.7	13.6	20.0	20.0	20.0	20.0	0.195
*Yr32*	13.8	47.4	20.0	27.1	13.6	4.20	0.00	25.0	10.7	0.096
*Yr64*	48.3	31.6	82.0	54.0	50.0	41.7	55.0	36.0	45.7	0.637
*Yr10*	41.4	10.5	40.0	30.6	4.50	4.20	0.00	11.0	4.90	0.017
*Yr15*	0.00	0.00	0.00	0.00	0.00	0.00	0.00	0.00	0.00	
*Yr17*	44.8	26.3	50.0	42.0	13.6	12.5	20.0	25.0	17.8	0.036
*Yr44*	58.6	42.1	50.0	50.2	68.2	87.5	50.0	64.3	67.5	0.072
*YrSp*	31.0	21.1	40.0	30.7	13.6	16.7	0.00	25.0	13.8	0.039
*YrTr1*	31.0	31.6	20.0	27.5	0.00	8.30	0.00	0.00	2.10	0.001
*Yr26*	17.2	16.8	20.0	18.0	4.50	8.30	0.00	11.1	6.00	0.005
*Yr29*	79.3	55.0	73.0	69.1	55.0	50.0	30.0	46.4	45.4	0.022
*YrJu4*	83.3	68.4	70.0	73.9	86.4	87.5	55.0	85.7	77.4	0.387
*YrKy2*	30.0	52.6	60.0	47.5	54.5	54.2	50.0	64.3	55.8	0.185
*YrRes*	93.3	78.9	70.0	80.7	68.2	58.3	70.0	82.1	69.7	0.115

**Table 3:** Unique virulence patterns (UVPs), average virulence complexity (AVC), relative virulence complexity (RVC), and virulence uniformity (VU) among populations of *Pst* in the western provinces of China.

Population	Total Isolates	UVP (%)	AVC	RVC	VU
SCNI	29	100	14.4	0.46	0.31
SCOI	22	100	10.6	0.34	0.36
YNNI	19	100	13.7	0.44	0.30
YNOI	24	100	12.4	0.40	0.26
GZNI	11	100	14.4	0.46	0.23
GZOI	10	100	11.5	0.37	0.27
GSOI	28	100	13.6	0.44	0.24

**Table 4:** Pairwise associations between virulence and avirulence in *Pst* isolates, at respective *Yr* loci.

*Yr* Gene Pair	Pathogen Alleles ^a^				Total	VV ^b^ Proportion	Association Type ^c^	*p*-Value ^d^
	AA	AV	VA	VV				
*Yr76 41*	88	10	11	34	143	0.24	+	<0.001
*Yr76 32*	93	6	35	9	143	0.06	+	0.016
*Yr76 10*	92	7	34	10	143	0.07	+	0.012
*Yr76 Sp*	86	14	30	13	143	0.09	+	0.038
*Yr76 Tr1*	91	8	34	10	143	0.07	+	0.026
*Yr50 41*	119	17	3	4	143	0.03	+	0.009
*Yr50 5*	130	2	7	4	143	0.03	+	<0.001
*Yr50 24*	110	26	3	4	143	0.03	+	0.035
*Yr50 10*	125	11	3	4	143	0.03	+	0.002
*Yr50 Sp*	114	22	3	4	143	0.03	+	0.021
*Yr50 Tr1*	125	11	3	4	143	0.03	+	0.002
*Yr50 26*	121	15	4	3	143	0.02	+	0.043
*Yr50 Ju4*	28	0	60	55	143	0.38	+/−	<0.001
*Yr50 Ky2*	69	67	0	7	143	0.05	+/−	0.014
*Yr41 10*	114	8	14	7	143	0.05	+	0.002
*Yr41 17*	98	24	7	14	143	0.10	+	<0.001
*Yr41 Ky2*	64	58	5	16	143	0.11	+/−	0.018
*Yr24 32*	101	27	3	12	143	0.08	+	<0.001
*Yr24 10*	105	8	23	7	143	0.05	+	0.017
*Yr24 26*	105	8	20	10	143	0.07	+	<0.001
*Yr32 26*	117	11	8	7	143	0.05	+	<0.001
*Yr17 Sp*	92	13	25	13	143	0.09	+	0.006
*Yr17 26*	98	7	27	11	143	0.08	+	0.001
*YrSp Tr1*	109	8	19	7	143	0.05	+	0.007
*YrSp 26*	107	10	18	8	143	0.06	+	0.005
*YrTr1 26*	117	11	8	7	143	0.05	+	<0.001

^a^ Number of virulent isolates (V) or avirulent (A), at respective *Yr* genes. ^b^ Proportion of isolates virulent to both *Yr* genes. ^c^ +, a positive association with AA or VV; +/−, an unclear association. ^d^ Pairs significantly different at *p* < 0.05 based on Fisher’s exact test are listed.

**Table 5:** Population genetic parameters among *Pst* populations.

*Pst* Population	*N*	*Na*	*Ne*	*I*	*uh*	*PPL* (%)
Old population	84	1.94	1.58	0.51	0.34	97
New population	59	1.97	1.53	0.48	0.32	97
Guizhou population	21	1.81	1.54	0.46	0.32	86
Sichuan population	51	2.00	1.54	0.49	0.33	100
Yunnan population	43	2.00	1.56	0.51	0.34	100
Gansu population	28	1.94	1.56	0.51	0.35	97
Mean	47.67	1.94	1.55	0.49	0.33	96

*N*, number of samples; *Na*, number of polymorphic alleles; *Ne*, number of effective alleles; *I*, Shannon information index; *uh*, unbiased diversity; *PPL*, percentage of polymorphic loci.

**Table 6:** Analysis of molecular variance (AMOVA) among provinces (Sichuan, Yunnan, Gansu, and Guizhou) and isolate types (old and new isolates) of *Pst* populations.

Source of Variation	Degree of Freedom	Sum of Squares	Component of Variations	Percentage of Variation (%)	*p*-Value
Among provinces	3	56.6	0.4	6	0.001
Within provinces	139	839.6	6.0	94	0.001
Total	142	896.3	6.4	100	
Between isolate groups	1	57.1	0.7	11	0.001
Within isolate groups	141	848.0	6.0	89	0.001
Total	142	905.1	6.8	100	

**Table 7:** Pairwise matrix showing the Nei’s genetic distance among provinces of the *Pst* populations.

	YN	GZ	SC	GS
YN				
GZ	0.06			
SC	0.03	0.06		
GS	0.04	0.06	0.10	

## Data Availability

The data utilized and/or analyzed in the present study can be obtained from the corresponding author upon reasonable request.

## Supplementary Materials

The following supporting information can be downloaded at: https://www.mdpi.com/article/10.3390/genes15050542/s1, Table S1: List of two sets of wheat differentials used to differentiate *Pst* pathotypes in China; Table S2: Distribution of *Pst* pathotypes in western provinces of China.
