# Supplementary material for: Comparative Analysis of Virulence and Molecular Diversity of Puccinia striiformis f. sp. tritici Isolates Collected in 2016 and 2023 in the Western Region of China
Source: Genes (Basel). 2024 Apr 25;15(5):542. doi: 10.3390/genes15050542 (PMC11121451; doi:10.3390/genes15050542)
Supplement: Supplementary file 1 [file genes-15-00542-s001.zip › genes-2930539-supplementary.pdf]

**Table S1**. List of two sets of wheat differentials used to differentiate *Pst* pathotypes in China

| | *Yr* single gene differentials | | Chinese differentials | | |
|---|---|---|---|---|---|
| No. | Name | *Yr* gene | Code | Name | *Yr* gene |
| 1 | Lemhi | *Yr21* | 1 | Trigo Eureka | *Yr6* |
| 2 | Tyee | *Yr76* | 2 | Fulhard | Unknown |
| 3 | CH223 | *Yr50* | 3 | Lutescens 128 | Unknown |
| 4 | Vasu | *Yr40* | 4 | Mentana | Unknown |
| 5 | CN19 | *Yr41* | 5 | Virgilio | *YrVir1*, *YrVir2* |
| 6 | PI181434 | *Yr45* | 6 | Abbondanza | Unknown |
| 7 | Suwon 11 | *YrSu* | 7 | Early Premium | Unknown |
| 8 | Avs/ID0377s NIL | *Yr43* | 8 | Funo | *YrA*,+ |
| 9 | Vocet R | *YrA* | 9 | Danish 1 | *Yr3* |
| 10 | TP1295 | *Yr25* | 10 | Jubilejina 2 | *YrJu1*, *YrJu2*, *YrJu3*, and *YrJu4* |
| 11 | AvSYr1 NIL | *Yr1* | 11 | Fengchan 3 | *Yr1* |
| 12 | AvSYr5 NIL | *Yr5* | 12 | Lovrin 13 | *Yr9*,+ |
| 13 | AvSYr6 NIL | *Yr6* | 13 | Kangyin 655 | *Yr1*, *YrKy1*, and *YrKy2* |
| 14 | Minister | *Yr3c* | 14 | Suwon 11 | *YrSu* |
| 15 | AvSYr7 NIL | *Yr7* | 15 | Zhong 4 | Unknown |
| 16 | AvSYr8 NIL | *Yr8* | 16 | Lovrin 10 | *Yr9* |
| 17 | AvSYr9 NIL | *Yr9* | 17 | Hybrid 46 | *Yr3b* and *YrH46* |
| 18 | AvSY24NIL | *Yr24* | 18 | *T.spelta* album | *Yr5* |
| 19 | AvSYr32NIL | *Yr32* | 19 | Guinong 22 | *Yr10* and *Yr26* |
| 20 | PI331260 | *Yr64* | | | |
| 21 | AvSYr10NIL | *Yr10* | | | |
| 22 | AvSYr15NIL | *Yr15* | | | |
| 23 | AvSYr17NIL | *Yr17* | | | |
| 24 | AvstZak NIL | *Yr44* | | | |
| 25 | AvSYrSPNIL | *YrSp* | | | |
| 26 | AvSYrTreslNIL | *YrTr1* | | | |
| 27 | AvSY26 NIL | *Yr26* | | | |
| 28 | AvSY29NIL | *Yr29* | | | |
| 29 | YrJu4 NIL | *YrJu4* | | | |
| 30 | YrKy2 NIL | *YrKy2* | | | |
| 31 | YrRes NIL | *YrRes* | | | |

**Table S2.** Distribution of *Pst* pathotypes in western provinces of China

| No. | Pathotype [a] | Yunnan | Guizhou | Sichuan | Gansu | Isolates/Mean | Frequency (%) |
|---|---|---|---|---|---|---|---|
| 1 | *V2, 4, 8, 14* | (2) | 1 (2)[b] | 1 (1) | | 7 | 5.0 |
| 2 | *V2, 4, 8, 12, 14, 16* | | | 5 | (1) | 6 | 4.2 |
| 3 | *V2, 8, 14* | 1 (1) | 1 | 1 (1) | | 5 | 3.5 |
| 4 | *V0* | (1) | (2) | | (2) | 5 | 3.5 |
| 5 | *V4, 14, 16* | | 1 | 3 | | 4 | 2.8 |
| 6 | *V2,14* | (2) | (1) | (1) | | 4 | 2.8 |
| 7 | *V2, 8, 14, 19* | (1) | (1) | (2) | | 4 | 2.8 |
| 8 | *V2, 4, 8, 10, 14, 19* | 2 | (1) | (1) | | 4 | 2.8 |
| 9 | *V2, V8* | | | (2) | (2) | 4 | 2.8 |
| 10 | *V2, 4, 14, 16* | | | 3 | | 3 | 2.1 |
| 11 | *V2, 3, 4, 6, 7, 8, 10, 11, 12, 14, 16, 19* | 1 | | 1 | | 2 | 1.4 |
| 12 | *V2, 10* | 1 | | 1 | | 2 | 1.4 |
| 13 | *V3, 7, 10, 19* | | 1 | (1) | | 2 | 1.4 |
| 14 | *V2, 4, 8, 11, 14,16* | 1 | 1 | | | 2 | 1.4 |
| 15 | *V2, 8, 14, 16* | 2 | | | | 2 | 1.4 |
| 16 | *V2, 8, 19* | | | (2) | | 2 | 1.4 |
| 17 | *V2, 7, 8, 14, 19* | (1) | | | (1) | 2 | 1.4 |
| 18 | *V2, 4, 7, 8, 14* | (1) | 1 | | | 2 | 1.4 |
| 19 | *V2* | | (1) | | (1) | 2 | 1.4 |
| 20 | *V19* | | | (1) | | 1 | 0.70 |
| 21 | *V12* | | 1 | | | 1 | 0.70 |
| 22 | *V14* | | | (1) | | 1 | 0.70 |
| 23 | *V8* | | | (1) | | 1 | 0.70 |
| 24 | *V2, 4* | | | | (1) | 1 | 0.70 |
| 25 | *V2, 3* | | | 1 | | 1 | 0.70 |
| 26 | *V7, 14* | | | | (1) | 1 | 0.70 |
| 27 | *V8, 19* | (1) | | | | 1 | 0.70 |
| 28 | *V2, 8, 17* | | (1) | | | 1 | 0.70 |
| 29 | *V7, 8, 10* | | | | (1) | 1 | 0.70 |
| 30 | *V2, 3, 14* | | | | (1) | 1 | 0.70 |
| 31 | *V2, 14, 16* | | | | (1) | 1 | 0.70 |
| 32 | *V8, 10, 14* | 1 | | | | 1 | 0.70 |
| 33 | *V2, 4, 5, 14* | | 1 | | | 1 | 0.70 |
| 34 | *V4, 7, 8, 12* | (1) | | | | 1 | 0.70 |
| 35 | *V6, 7, 8, 14* | | | | (1) | 1 | 0.70 |
| 36 | *V2, 3, 7, 10* | | | | (1) | 1 | 0.70 |
| 37 | *V2, 4, 8, 16* | (1) | | | | 1 | 0.70 |
| 38 | *V2, 8, 10, 14* | (1) | | | | 1 | 0.70 |
| 39 | *V2, 8, 10, 19* | | | (1) | | 1 | 0.70 |
| 40 | *V2, 4, 14, 19* | | | (1) | | 1 | 0.70 |
| 41 | *V2, 7, 8, 14* | (1) | | | | 1 | 0.70 |
| 42 | *V2, 5, 7, 19* | | | (1) | | 1 | 0.70 |
| 43 | *V2, 3, 7, 13, 16* | | | | (1) | 1 | 0.70 |
| 44 | *V2, 7, 8, 13, 19* | | (1) | | | 1 | 0.70 |
| 45 | *V2, 4, 6, 8, 14* | (1) | | | | 1 | 0.70 |
| 46 | *V2, 6, 10, 14, 19* | | | | (1) | 1 | 0.70 |
| 47 | *V2, 4, 8, 10, 14* | | | 1 | | 1 | 0.70 |
| 48 | *V2, 4, 8, 10, 11* | (1) | | | | 1 | 0.70 |
| 49 | *V2, 6, 8, 16, 19* | | | (1) | | 1 | 0.70 |
| 50 | *V2, 7, 11, 14, 19* | (1) | | | | 1 | 0.70 |
| 51 | *V2, 3, 4, 8, 16* | | | | (1) | 1 | 0.70 |
| 52 | *V2, 4, 11, 12, 14* | (1) | | | | 1 | 0.70 |
| 53 | *V2, 8, 12, 14, 19* | | | (1) | | 1 | 0.70 |
| 54 | *V2, 3, 4, 10, 11, 12* | | | (1) | | 1 | 0.70 |
| 55 | *V2, 3, 4, 8, 10, 14* | | | | (1) | 1 | 0.70 |

**Continued Table S2**

| No. | Pathotype | Yunnan | Guizhou | Sichuan | Gansu | Isolate/Mean | Frequency (%) |
|---|---|---|---|---|---|---|---|
| 56 | *V2, 3, 4, 8, 14,19* | | | | (1) | 1 | 0.70 |
| 57 | *V3, 4, 8, 11, 14, 19* | (1) | | | | 1 | 0.70 |
| 58 | *V2, 3, 6, 7, 8, 16* | | | (1) | | 1 | 0.70 |
| 59 | *V3, 4, 7, 8, 10, 14* | | | (1) | | 1 | 0.70 |
| 60 | *V2, 4, 7, 8, 12, 14* | | | | (1) | 1 | 0.70 |
| 61 | *V2, 3, 4, 7, 8, 14* | (1) | | | | 1 | 0.70 |
| 62 | *V2, 4, 7, 12, 14, 17* | | | | (1) | 1 | 0.70 |
| 63 | *V2, 8, 10, 11, 17, 19* | | | | (1) | 1 | 0.70 |
| 64 | *V2, 3, 4, 11, 12, 14* | | | 1 | | 1 | 0.70 |
| 65 | *V1, 2, 4, 6, 8, 10, 14* | (1) | | | | 1 | 0.70 |
| 66 | *V2, 4, 6, 7, 8, 10,* 14 | | | 1 | | 1 | 0.70 |
| 67 | *V2, 4, 7, 8, 10, 11, 14* | | | 1 | | 1 | 0.70 |
| 68 | *V1, 2, 3, 4, 7, 8, 16* | (1) | | | | 1 | 0.70 |
| 69 | *V2, 4, 6, 8, 12, 14, 16* | 1 | | | | 1 | 0.70 |
| 70 | *V2, 4, 6, 8, 11, 14, 16,* | 1 | | | | 1 | 0.70 |
| 71 | *V2, 4, 6, 7, 8, 14, 16,* | | | | (1) | 1 | 0.70 |
| 72 | *V2, 4, 8, 11, 12, 14, 16, 17* | | 1 | | | 1 | 0.70 |
| 73 | *V2, 3, 6, 8, 11, 14, 16, 18* | | | | (1) | 1 | 0.70 |
| 74 | *V2, 4, 6, 7, 8, 12, 14, 16* | 1 | | | | 1 | 0.70 |
| 75 | *V2, 3, 4, 10, 11, 12, 14, 16* | | | 1 | | 1 | 0.70 |
| 76 | *V2, 3, 4, 8, 10, 14, 16, 19* | | | 1 | | 1 | 0.70 |
| 77 | *V2, 3, 4, 7, 10, 13, 14, 19* | | 1 | | | 1 | 0.70 |
| 78 | *V2, 4, 6, 8, 9, 12, 14, 16* | (1) | | | | 1 | 0.70 |
| 79 | *V2, 4, 6, 8, 11, 12, 14, 16* | | | 1 | | 1 | 0.70 |
| 80 | *V2, 4, 7, 8, 10, 14, 16, 19* | | | | (1) | 1 | 0.70 |
| 81 | *V2, 3, 7, 10, 12, 13, 14, 19* | 1 | | | | 1 | 0.70 |
| 82 | *V2, 3, 4, 8, 10, 16, 17, 19* | 1 | | | | 1 | 0.70 |
| 83 | *V1, 6, 7, 8, 9, 12, 14, 16, 17* | 1 | | | | 1 | 0.70 |
| 84 | *V2, 3, 4, 7, 8, 9, 10, 11, 14* | | | 1 | | 1 | 0.70 |
| 85 | *V2, 3, 4, 6, 7, 8, 10, 14, 16,* | | | | (1) | 1 | 0.70 |
| 86 | *V2, 4, 5, 6, 8, 12, 14, 16, 17* | | | | (1) | 1 | 0.70 |
| 87 | *V2, 3, 4, 8, 9, 10, 13, 14, 16* | 1 | | | | 1 | 0.70 |
| 88 | *V2, 3, 4, 5, 7, 8, 10, 14, 17, 19* | | 1 | | | 1 | 0.70 |
| 89 | *V2, 4, 5, 6, 7, 8, 11, 12, 14, 16* | | | (1) | | 1 | 0.70 |
| 90 | *V2, 3, 7, 9, 11, 13, 16, 17, 18, 19* | 1 | | | | 1 | 0.70 |
| 91 | *V2, 4, 5, 7, 9, 11, 12, 14, 16, 17* | | | 1 | | 1 | 0.70 |
| 92 | *V2, 3, 4, 7, 8, 10, 12, 13, 14, 16* | | | | (1) | 1 | 0.70 |
| 93 | *V2, 3, 4, 6, 7, 8, 9, 10, 11,.14,16* | | | 1 | | 1 | 0.70 |
| 94 | *V2, 3, 4, 6, 7, 8, 9, 10, 14, 16, 19* | | | 1 | | 1 | 0.70 |
| 95 | *V2, 3, 4, 6, 7, 8, 9, 10, 12, 13, 14, 16* | | | 1 | | 1 | 0.70 |
| 96 | *V2, 3, 4, 5, 7, 8, 9, 10, 11, 12, 14, 16* | | | 1 | | 1 | 0.70 |
| 97 | *V2, 3, 4, 5, 6, 7, 8, 9, 11, 12, 14, 16, 17, 19* | 1 | | | | 1 | 0.70 |
| 98 | *V1, 2, 3, 4, 5, 6, 7, 8, 9, 10, 11, 12, 13, 14, 16, 17* | 1 | | | | 1 | 0.70 |
| | Total | 19 (23) | 11 (10) | 29 (23) | (28) | 143 | 100 |
| | *H* [c] | 3.6 | 2.8 | 3.5 | 3.6 | 3.4 | |

[a] Corresponding virulence (*V1-V19*) to the 19 Chinse differentials: *V0* = avirulent to all differentials; *V1* = Trigo Eureka (*Yr6*); *V2* = Fulhard (unknown); *V3* = Lutescens128 (unknown); *V4* = Mentana (unknown); *V5* = Virgilio (*YrVir1* and *YrVir2*); *V6* = Abbondanza (unknown); *V7* = Early premium (unknown); *V8* = Funo (*YrA,+)*; *V9* = Danish 1 (*Yr3*); *V10* = Jubilejina 2 (*YrJu1, YrJu2, YrJu3* and *YrJu4*); *V11* = Fengchan3 (*Yr1*); *V12* = Lovrin 13 (*Yr9,+*); *V13* = Kangyin 655 (*Yr1, YrKy1* and *YrKy2*); *V14* = Suwon 11 (*YrSu*); *V15* = Zhong 4 (unknown); *V16* = Lovrin 10 (*Yr9*); *V17* = Hybrid 46 (*Yr3b* and *Yr4b*); *V18* = *T. spelta* var album (*Yr5*); and *V19* = Guinong 22 (*Yr26*). [b] old and new isolates are shown inside and outside parentheses, respectively. [c] Shannon-Wiener pathotype diversity index (*H*) for the 143 *Pst* isolates.
